# Genomic characterization and related functional genes of γ- poly glutamic acid producing *Bacillus subtilis*

**DOI:** 10.1186/s12866-024-03262-z

**Published:** 2024-04-15

**Authors:** Jiayue Zhu, Xue Wang, Jianan Zhao, Fang Ji, Jun Zeng, Yanwen Wei, LiLi Xu, Guoying Dong, Xingyuan Ma, Chengmin Wang

**Affiliations:** 1 State Key Laboratory of Bioreactor Engineering, East China University of Science and Technology, Shanghai, 200237 China; 2 Guangdong key Laboratory of Wild Animal Conservation and Utilization, Institute of Zoology, Guangdong Academy of Science, Guangzhou, 510260 China; 3 Union Biology (Shanghai) Co., Ltd, Shanghai, 201100 China; 4 College of Global Change and Earth System Science, Faculty of Geographical Science, Beijing Normal University, Beijing, 100875 China

**Keywords:** γ- poly glutamic acid (γ-PGA), *Bacillus subtilis*, Genomics analysis, Functional genes

## Abstract

γ- poly glutamic acid (γ-PGA), a high molecular weight polymer, is synthesized by microorganisms and secreted into the extracellular space. Due to its excellent performance, γ-PGA has been widely used in various fields, including food, biomedical and environmental fields. In this study, we screened natto samples for two strains of *Bacillus subtilis* N3378-2at and N3378-3At that produce γ-PGA. We then identified the γ-PGA synthetase gene cluster (PgsB, PgsC, PgsA, YwtC and PgdS), glutamate racemase RacE, phage-derived γ-PGA hydrolase (PghB and PghC) and exo-γ-glutamyl peptidase (GGT) from the genome of these strains. Based on these γ-PGA-related protein sequences from isolated *Bacillus subtilis* and 181 *B. subtilis* obtained from GenBank, we carried out genotyping analysis and classified them into types 1–5. Since we found *B. amyloliquefaciens* LL3 can produce γ-PGA, we obtained the *B. velezensi*s and *B. amyloliquefaciens* strains from GenBank and classified them into types 6 and 7 based on LL3. Finally, we constructed evolutionary trees for these protein sequences. This study analyzed the distribution of γ-PGA-related protein sequences in the genomes of *B. subtilis, B. velezensi*s and *B. amyloliquefaciens* strains, then the evolutionary diversity of these protein sequences was analyzed, which provided novel information for the development and utilization of γ-PGA-producing strains.

**Supplementary Information:**

The online version contains supplementary material available at 10.1186/s12866-024-03262-z.

## Introduction

Poly-γ-glutamic acid (γ-PGA) is a polymer synthesized by microorganisms and secreted outside of their cells. It has a molecular weight range of 100 to 1000 kDa. Glutamic acid monomer is a high molecular polyamino acid polymer formed by condensation of the γ-carboxyl group and α-amino group [1, 2]. γ-PGA was originally discovered in the traditional Japanese food natto, which is produced by fermenting soybeans with *Bacillus subtilis* subsp. *natto*, and the stickiness of natto mainly results from this polymer [3]. γ-PGA is water-soluble, biodegradable, and edible, which enables it to be used in food and drugs, cosmetics, medical adhesives, and water treatment [4]. The application of γ-PGA corresponds with the development direction of green environmental protection, and this biomacromolecular polymer will have a broader development space in the future. γ-PGA can be stored as extracellular nutrients, providing microorganisms with survival needs during critical periods [5, 6], preventing the invasion of bacteriophages, and wrapping metal ions in high salt environments to protect cells [7].

γ-PGA is mainly produced by several *Bacillus* species, including *B. subtilis*, *B. licheniformis, B. amyloliquefaciens* and *B. megaterium*. Among them, *B. subtilis* CGMCC 0833 [8] was reported to produce 34.4 g/L γ-PGA after medium optimization, and under optimal fermentation conditions, the production of γ-PGA by *B. subtilis* natto is positively correlated with nattokinase activity, reaching a maximum yield of 358.5 g/kg after 36 h [9]. . The genetically engineered strain *B. tequilensis* BL01 Δ*pgdS*Δ*ggt*Δ*sucA*Δ*gudB*:P43-*citZ*-*pyk*, when cultured in a fermentation vessel containing 10 g/L sodium citrate, exhibited a γ-PGA titer of 25.3 ± 0.8 g/L and a productivity of 0.84 g/L/h. This represents a 2.17-fold increase in efficiency compared to the wild-type strain [10]. The γ-PGA production of *B. amyloliquefaciens* LL3 reached 7.12 g/L after a double deletion of *pgdS* and *cwlO* [11]. In *Bacillus* strains, γ-PGA synthesis is carried out by a transmembrane enzyme complex, which is encoded by four genes, *pgsB*, *pgsC*, *pgsA* and *pgsE*. And they were first identified in *B. subtilis* [12, 13]. They are involved in converting D- and L-glutamic acid into γ-PGA in an ATP-dependent reaction. PgsB and PgsC are responsible for γ-PGA polymerization, whereas PgsA is responsible for the inclusion of L-glutamic acid monomers and further transport of γ-PGA through the cell membrane [13–15]. Some studies have investigated the significance of the *pgsB*, *pgsC* and *pgsA* genes, whereas some researchers have determined that the *pgsE* gene is nonessential [8, 16]. YwtC is a leading small protein, and it may function to encode the *pgdS* gene. The γ-PGA productivity of *B. subtilis* subsp. *chungkookjang* could be increased by the PgsE enzyme in the presence of Zn^[2+ [17]]^ But the function of YwtC remains unknown.

However, to convert L-glutamic acid into D-glutam, two homologs of the glutamate racemase genes (*racE/glr* and *yrpC*) have been identified [18, 19], and they can encode RacE, which is highly preferable for converting L-glutamate to D-glutamate [20], but they are not responsible for γ-PGA synthesis [5]. There are also certain enzymes that can degrade γ-PGA. The *pgdS* gene, which is located downstream of the pgs operon, could cleave γ-PGA between D- and L-glutamic acid [21–23]. γ-Glutamyl transpeptidase (GGT, EC 2.3.2.2) also participates in γ-PGA degradation and removes glutamic acid monomers from the N-terminal side of γ-PGA [5, 6]. *B. subtilis* utilizes either glycolysis or the tricarboxylic acid cycle for its biochemical oxidation. It metabolizes various carbon sources present in the culture medium and synthesizes bioprecursors bearing glutamic acid residues [24]. The γ-glutamyl kinase encoded by *pgsB* phosphorylates glutamic acid to produce γ-glutamyl phosphate, initiating the biosynthetic pathway of γ-PGA. On the other hand, the γ-glutamyl phosphate reductase encoded by *pgsC* gene reduces γ-glutamyl phosphate back to the crucial intermediate L-glutamate-γ-semialdehyde of this pathway [3]. Additionally, the glutamate racemase encoded by the *pgsA* gene facilitates the conversion of L-glutamate-γ-semialdehyde to D-glutamate-γ-semialdehyde [2]. Ultimately, the multifunctional enzyme encoded by *pgsE*, possessing glutamate racemase and glutamate-5-semialdehyde dehydrogenase activities, catalyzes the conversion of D-glutamate-γ-semialdehyde to L-glutamate, thereby completing the synthesis of γ-PGA, which is then released extracellularly [25]. The synthesis of γ-PGA is an ATP-dependent process, and the synthesis of bioprecursors containing glutamic acid residues conveniently provides energy for this process [24]. Later, it was found that four unannotated *B. subtilis* gene products were derived from integrated prophages, and they were renamed PghB, PghC, PghL and PghZ [7]. To date, most of the research on γ-PGA-producing strains has focused on improving their production of γ-PGA and studying their biological function in γ-PGA synthesis [8, 26], and only a handful of studies have explored genomics-based strategies. Very few genome-associated studies have been conducted on γ-PGA-producing strains or the mechanisms underlying γ-PGA synthesis. Additionally, the role of γ-PGA-related enzymes in influencing the secretion of γ-PGA also remains largely unknown.

To address these issues, we isolated two γ-PGA producing *B. subtilis* strains from natto and obtained genome sequences of 181 *Bacillus subtilis*, 175 *Bacillus velezensis* and 55 *Bacillus amyloliquefaciens* strains from GenBank to carry out genome typing based on these γ-PGA-related protein sequences since they have been identified in 181 *Bacillus subtilis*, 175 *Bacillus velezensis* and 55 *Bacillus amyloliquefaciens* obtained from GenBank. Finally, we constructed phylogenetic trees to reveal the evolutionary diversity and differences in the amino acid sites of γ-PGA-related protein sequences.

## Materials and methods

### Isolation of γ-PGA producing *Bacillus* sp. strains

γ-PGA-producing strains were isolated from natto in Hokkaido, Japan. To grow the *Bacillus* isolates, Mueller-Hinton Broth medium (MHB; 5 g/L beef paste powder, 1.5 g/L starch and 17.5 g casein hydrolysate) was used as the basal medium. Samples of 1 g natto were diluted in 10 mL of sterilized distilled water and spread on MH(A) medium. Colonies with different microscopic shapes were selected and transferred to MH(B) medium and then incubated at 37℃ with 200 rpm agitation for 12 h. The strains were then stored in MH(B) medium with sterile glycerol (20%, v/v) at -80℃.

### Screening and identification for γ-PGA producing *Bacillus* sp. strains

The *Bacillus* isolates were spread onto isolation medium(glucose 20 g/L, yeast extract 25.0 g/L, MgSO_4_ · 7H_2_O 0.5 g/L, K_2_HPO_4_ 1.0 g/L, pH 7.0), and after incubation at 37°C for 20 h, sticky clones were picked and transferred into 200 mL of fermentation medium containing 15 g/L glucose, 15 g/L tryptone, 25.0 g/L yeast extract, 0.6 g/L MgSO_4_·7H_2_O, 1.0 g/L K_2_HPO_4_ and 0.1 g MnSO_4_ (pH 7.0) in a 500-mL flask. The strains were incubated at 35°C with 200 rpm agitation for 22 h. The 16S rDNA sequences of selected isolates were amplified by PCR using universal primers 27F (5’-AGAGTTTGATCCTGGCTCAG-3’) and 1492R (5’-CGGTTACCTTGTTACGACTT-3’) [27], which were described in a previous study. The PCR products were then sequenced and 16 S rDNA sequences of other *Bacillus* spp. were obtained from the National Center for Biotechnology Information (NCBI, https://submit.ncbi.nlm.nih.gov/) website, and their homology was analyzed using the Basic Local Alignment Search Tool (BLAST). A phylogenetic tree based on the 16 S rDNA genes was constructed using the maximum likelihood method in MEGA-X.

### Purification and identification of γ-PGA

The pH value was maintained at 4.0 throughout fermentation by adding 2 M HCl, and after 22 h fermentation, the fermentation medium was centrifuged at 15,100 RCF for 15 min. The supernatant was collected, and 3 volumes of pure ethanol were added to the mixture, which was then refrigerated overnight at 4 °C. Next, the pellet containing γ-PGA was dissolved in deionized water and purified using a dialysis bag with a molecular weight cutoff of 8000–14,000 Da. Finally, the γ-PGA solution was dried using a vacuum freeze dryer, and the resulting product was used for further analysis. The concentration of γ-PGA can be measured using the UV spectroscopy method as reported previously [28]. For this method, a standard γ-PGA solution (200 µg/mL) was prepared. In particular, the solution was mixed with deionized water at pH 7.0 and stored at 4 °C. Initially, absorption spectra (190–400 nm) were recorded for the standard γ-PGA solution (200 µg/ml). Subsequently, the γ-PGA content of the crude sample was assayed by UV spectrophotometry between 190 and 400 nm. The maximum absorption wavelength of γ-PGA can be determined at 216 nm using a UV spectrophotometer.

The purified polymer and a γ-PGA standard (0.2 g) were hydrolyzed by HCl (6 mol/L) at 110 °C for 22 h in a sealed and evacuated tube, neutralized with 6 M NaOH and each analyzed by 0.2% ninhydrin using φ 50-mL culture tubes. Then, the L-glutamate standard (0.1000 g) was used to construct a standard curve. The optical density (OD) of the products was detected using a UV detector at a wavelength of 570 nm. The γ-PGA content was calculated using the following equation:

$$\begin{aligned} & \gamma-PGA\;content\;\left({\mathbf{\mu} }{\mathbf{g}}\right)\\ &\;=\frac{Glutamic\;Acid\;Content\;of\;Fermentation\;Sample \left(\varvec{\mu }\varvec{g}\right)}{(\mathbf{G}/\mathbf{W})\times 100\mathbf{\%}}\end{aligned}$$

where G is the content of glutamic acid (µg) obtained from the standard curve, and W is the amount of the sample hydrolysate determined to be equivalent to the sample (µg).

### Genome sequencing and annotation of γ-PGA-producing isolates

N3378-2at and N3378-3At were sent to sequence the whole genome (Beijing Biomark Biotechnology Co., Ltd.). Whole genome sequencing (WGS) was performed based on the Nanopore sequencing counting platform. The entire experimental protocol was sequenced according to the ONT standard method, high-quality genomic DNA was extracted, and the purity, concentration and integrity were checked by Nanodrop, Qubit and 0.35% agarose gel electrophoresis [29, 30]. Through BluePippin, large fragments of DNA were recovered. The SQK-LSK109 ligation kit was used to construct the library, and upper-level sequencing was performed. After quality control of raw data, genome assembly and error correction, Prodigal software was used to predict the coding genes of the assembled genome and perform functional annotation and comparative genomic analysis of the genes. Blast [31] sequence alignment was performed on the predicted gene sequences with major functional databases such as COG [32], KEGG [33], Swiss-Prot [34], TrEMBL [34] and Nr [35]. The genomes of both strains are annotated using the same databases. According to the comparison results of the Nr database, Blast2GO software [36] was used to perform the functional annotation of GO [37], and the application software hmmer [38] was used to perform the functional annotation based on the Pfam [39] database. In addition, gene functional annotation analysis, such as COG, KEGG and GO metabolic pathways and functional enrichment analysis, all sequenced bacterial whole genome sequences were registered in the DNA sequence database established by the National Center for Biotechnology Information (GenBank, https://submit.ncbi.nlm.nih.gov/).

### Distribution of γ-PGA relative genes of *Bacillus sp*. isolates genome and gene typing

This study focused on genome analysis of strains of *B. subtilis*, *B. velezensis*, and *B. amyloliquefaciens* isolated from various regions and deposited in the GenBank database between 1990 and 2021. After excluding incomplete or ambiguously background sequences, a total of 183 strains of *B. subtilis*, 175 strains of *B. velezensis*, and 55 strains of *B. amyloliquefaciens* were used for subsequent genotyping analysis (Supplementary file 1). Fasta format sequences of γ-PGA synthetase gene cluster (*pgsB*, *pgsC*, *pgsA*, *ywtC*, and *pgdS*), phage-derived γ-PGA hydrolases (*pghB*, *pghC*, *pghL*, and *pghZ*), glutamate racemase (*racE*), and γ-glutamyltransferase (*ggt*) were extracted from the annotation files provided by GenBank. Based on the presence and homology of these genes in the genomes, they were divided into different gene types. Subsequently, each gene type was further subdivided into different subtypes based on their arrangement, length, transcription direction, and distance in the genome, and labeled with different numbers. Notably, *B. amyloliquefaciens* LL3 was chosen as the reference sequence for gene typing due to being the first reported strain carrying the non-glutamate-dependent γ-PGA synthetase gene cluster *pgsBCA* [40]. Visualization of all different genotypes in this study will be implemented using the Illustrator for Biological Sequences (IBS) tool [41].

### Phylogenetic analysis based on γ-PGA synthetase genes

To analyze the sites of amino acid diversity in all *B. subtilis* genomes, the N3378-2at strain was selected as a reference strain since it was able to produce higher yield of γ-PGA compared to the N3378-3At strain, and the amino acid sequences of PgsB, PgsC, PgsA, PgdS, PghB, PghC, RacE, and GGT were used as target alignment sequences. An alignment of each sequence was generated using the ClustalW program [42] available at http://clustalw.genome.ad.jp/. The amino acid sequences of these γ-PGA-related enzymes were subjected to the maximum likelihood method with 1000 bootstraps using MEGA-X version.

## Results

### Isolation and identification of γ-PGA-producing *B. subtilis*

**Fig. 1:**
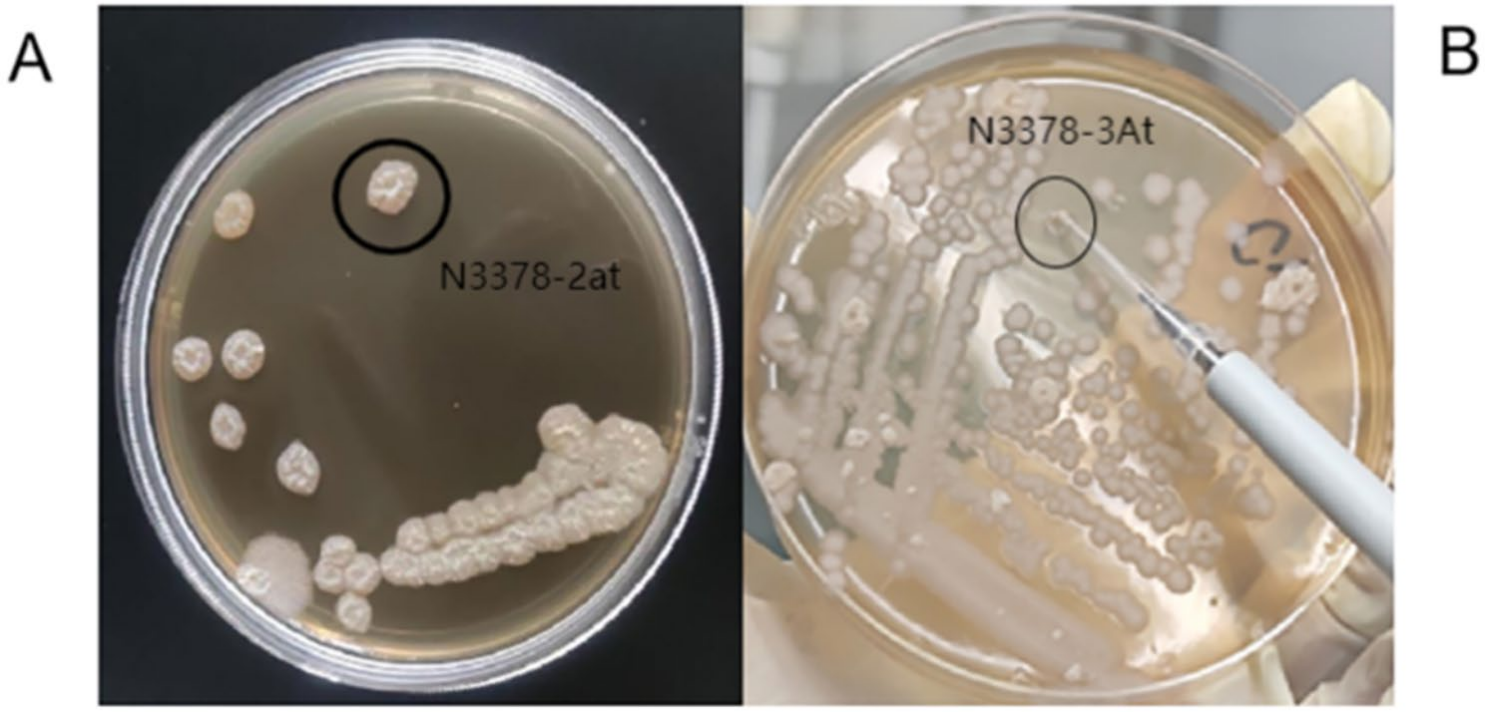
γ-PGA producing *Bacillus* spp. selected by isolation medium. (**A**) Appearance of N3378-2at strain. (**B**) Appearance of N3378-3At strain

To screen for isolates that can produce γ-PGA, two isolates with sticky colonies were selected from the isolation medium. These strains were identified as N3378-2at (Fig. 1A) and N3378-3At (Fig. 1B). Phylogenetic tree was constructed with maximum likelihood method based on the evolutionary distance calculated from 1,000 replicates has showed 4 distinct clusters **(not shown)**, which were separated on a scale of 0.01 nucleotide substitution. The accession numbers of *Bacillus* spp. and were shown in Supplementary file 1. For these strains, NRC20 showed similarities with NBRC 101,234, NBRC 15,535 showed similarities with PD-A10. N3378-2at, N3378-3At, *B. subtilis* NBRC 13,719, *B. subtilis* JCM 1465 and *B. subtilis* BCRC 10,255 with 95.93% of similarity percentage in in cluster 4.

### Identification and quantification of γ-PGA

**Fig. 2:**
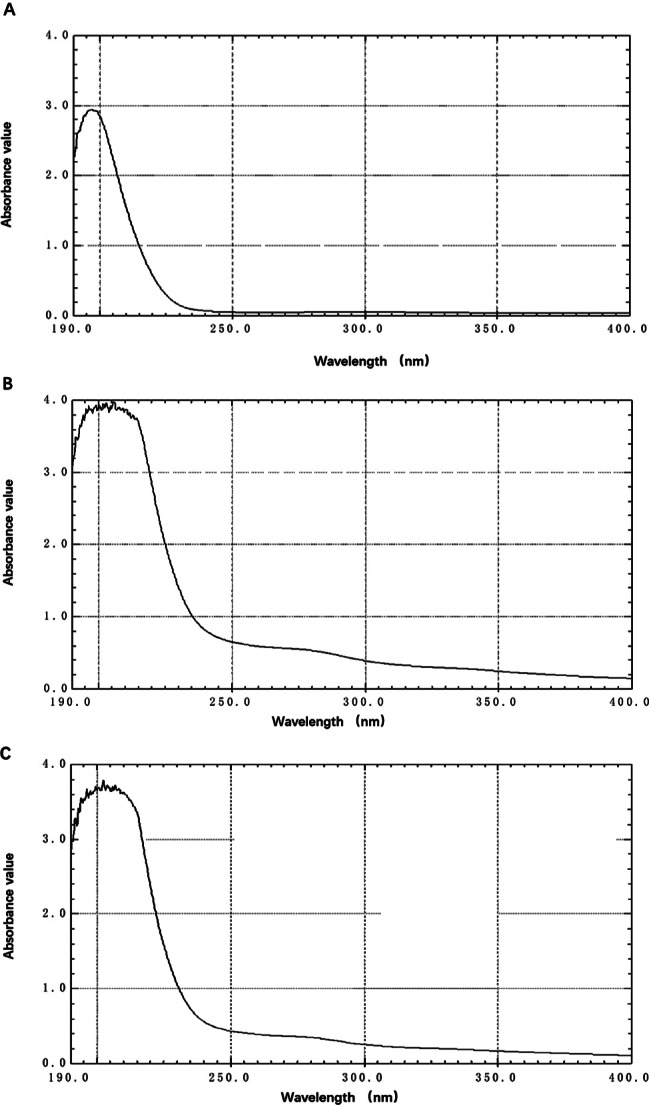
UV−visible absorption spectrum of γ-PGA polymers. (**A**) γ-PGA standard, (**B**) N3378-2at strain and (**C**) N3378-3At strain

The ability to identify and quantify γ-PGA in an easy and reliable manner is significant when analyzing natural samples. We analyzed the products by the UV spectrophotometric method. In addition, some interfering substances are found in the products that may affect the accuracy of γ-PGA determination. The maximum absorption wavelength of the γ-PGA standard was 198 nm, and the peak value was 2.98 (Fig. 2A). The maximum absorption wavelength of the fermentation product of N3378-2at was 196 nm, and the peak value was 3.81 (Fig. 2B). The maximum absorption wavelength of the fermentation product of N3378-3At is 192, and the peak is 3.89 (Fig. 2C). Therefore, the crude products of N3378-2at and N3378-3At have only one peak, which is very similar to the ultraviolet maximum absorption wavelength of poly-γ-glutamic acid. The fermentation products of N3378-2at and N3378-3At were both γ-PGA.

The concentration of L-glutamic acid was determined by the ninhydrin colorimetric method. Taking the concentration of the L-glutamic acid standard solution as the abscissa and the absorbance (A570 nm) as the abscissa, the L-glutamic acid standard curve was drawn, and the standard curve was simulated by regression. The obtained equation was y = 359.87x + 250.1, R^2^ = 0.978. The L-glutamic acid standard curve had good linearity. After the fermentation broths of strains N3378-2at and N3378-3At were purified and freeze-dried, the fermentation samples obtained were weighed. The weights of the fermentation crude products of N3378-2at and N3378-3At were 0.71 g and 0.681 g, respectively. The hydrolyzed content of the γ-PGA standard measured in the experiment was 1600 µg. According to the formula γ-PGA content=(G/W)×100%, the content of γ-PGA in the lyophilized fermentation products of the N3378-2at and N3378-3At strains was obtained. (Table 1).

**Table 1:** Yield of γ-PGA of *B. subtilis* strains N3378-2at, N3378-3At

Strain	OD_600_ value	L-glutamic acid content (µg)	γ-PGA content(g)	Yield
N3378-2at	1.881	927.02	0.4114	2.0568 g/L
N3378-3At	1.437	767.23	0.3266	1.6328 g/L

### Characterization of γ-PGA-related enzyme gene clusters in *Bacillus* spp. genomes

Whole-genome sequencing of *B. subtilis* N3378-2at and N3378-3At revealed the presence of the γ-PGA synthetase gene cluster (PgsB, PgsC, PgsA, YwtC, and PgdS), glutamate racemase RacE, phage-derived γ-PGA hydrolase (PghB, PghC, and PghL), and exo-γ-glutamyl peptidase (GGT). Further alignment analysis utilizing the γ-PGA synthetase-related protein sequences from *B. subtilis* N3378-2at revealed that, apart from 2 strains of *B. subtilis* and 3 strains of *B. amyloliquefaciens*, PgsB, PgsC, PgsA, YwtC, PgdS, RacE, PghB, PghC, and GGT were distributed across all other 181 strains of *B. subtilis*, 175 strains of *B. velezensis*, and 52 strains of *B. amyloliquefaciens genomes*, whereas the γ-PGA synthetase genes associated with bacteriophage genomes, pghL, and pghZ, were exclusively present in the *B. subtilis* genome (181 and 57 hits, respectively). The majority of *Bacillus* spp. isolates carry either the *pgs* gene cluster or the phage-derived *pgh* gene cluster, suggesting that *Bacillus* spp. populations serve as potential excellent producers of γ-PGA.

### Genotype classification based on γ-PGA-related protein sequences

Bacterial Genome-wide association study (BGWAS) is conducive to finding genetic differences significantly related to phenotype from huge genomes, thus clarifying the genetic mechanism of phenotype [43]. Thus, we carried out genotyping analysis of γ-PGA-related protein sequences based on *B. subtilis*, *B. velezensi*s and *B. amyloliquefaciens* strains. Based on the presence of specific genes in the genome, the 183 strains of *B. subtilis* were categorized into five genotypes, namely, G1 ∼ G5, which were further divided into several subtypes based on gene order, distance, and direction. The G1 genotype strains were divided into nine subtypes (SubGenotype, SG1.1 ∼ SG1.9), while the G2 genotype strains could be further divided into SG2.1 ∼ SG2.8. The G3 genotype was divided into SG3.1 ∼ SG3.4, whereas only one *B. subtilis* strain belonged to each of the G4 and G5 genotypes. Furthermore, after homology comparison was performed, the 175 strains of *B. velezensis* and 55 strains of *B. amyloliquefaciens* were categorized into two genotypes, G6 and G7, in which G6 was further subdivided into SG6.1 ∼ SG6.10. Among the *B. subtilis* strains, 48 belonged to genogroup 1 (subtype 1). It is evident that the genotypes of *B. subtilis* are primarily SG1.1 and SG2.1, in which SG2.1 is the most common genotype, with 94 strains, followed by SG1.1 with 49 strains. In contrast, the genotypes of *B. velezensis* and *B. amyloliquefaciens* strains primarily belong to G6 and G7, in which the main category is SG6.1, comprising 173 strains.

**Fig. 3:**
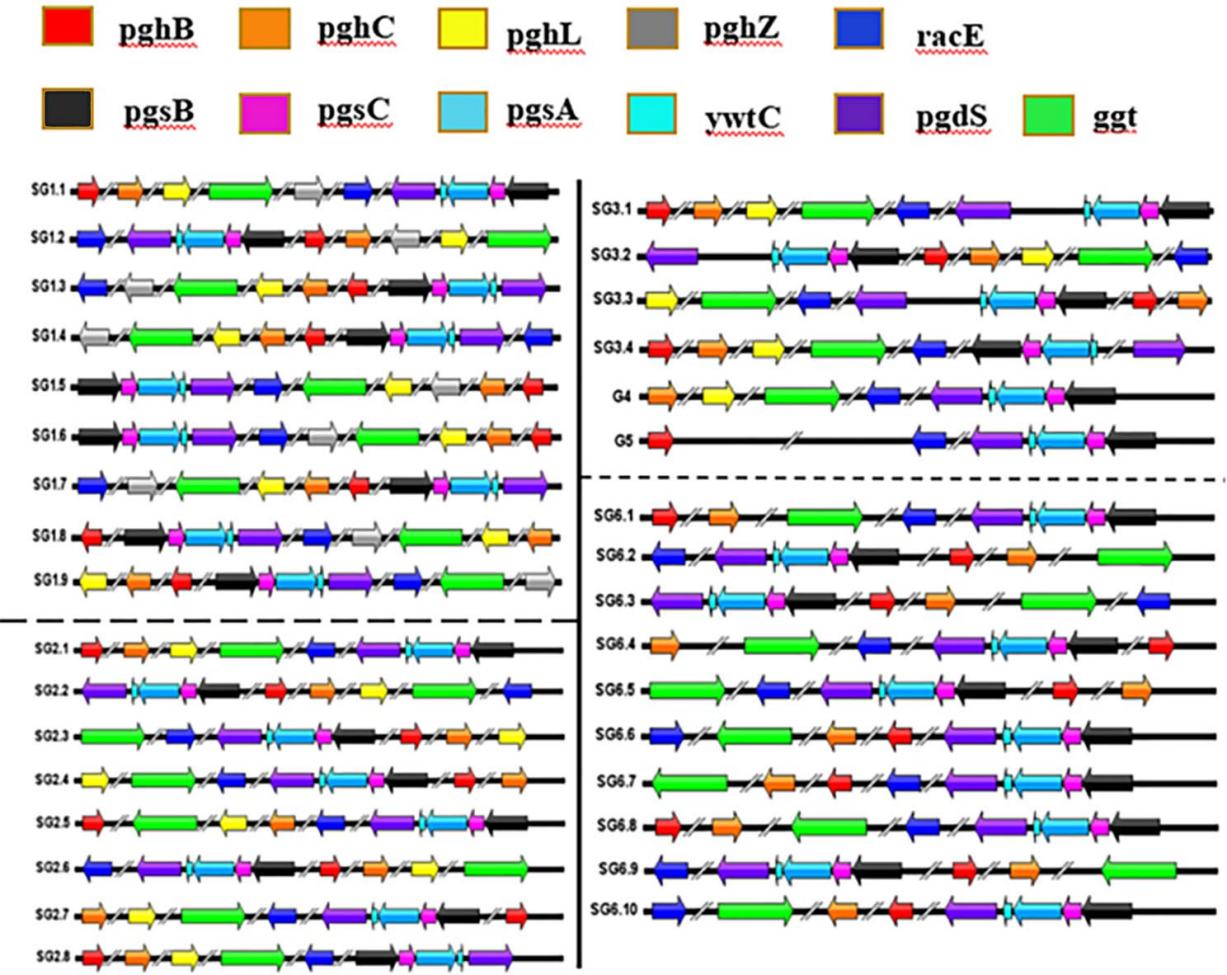
Illustration of genotyping of *Bacillus* sp. Strains. Among these *Bacillus* spp. strains, *B. subtilis* strains are grouped into genotypes 1–5, *B. amyloliquefaciens* and *B. velezensis* strains are grouped into genotypes 6 and 7

The following results are described through IBS online mapping (Fig. 3). The genotype 1 includes the genes *pghB*, *pghC*, *pghL*, *pghZ*, *ggt*, *racE*, *pgsB*, *pgsC*, *pgsA*, *pgsE* and *pgdS*. The strains N1142-3at, N1282-4at, N1108-5at and N1303-2Ay cannot secrete γ-PGA, and they belong to the genotypes SG1.6, SG1.7, SG1.8 and SG1.9, respectively. The gene type and ordering method of the N1142-3at genome are the same as those of N1142-3at (SG1.3), but the *pgdS* and *racE* of N1142-3at and SRCM102754 are in opposite directions of gene transcription, so the N1142-3at strain is listed as the SG1.6 genotype. The same is observed for the genotypes of the N1282-4at strain compared with the SG1.1 strain. The gene transcription direction of the γ-PGA synthetase gene cluster of SG1.1, SG1.3 and SG1.4 is opposite to that of the remaining γ-PGA enzymes. The γ-PGA synthetase gene cluster and pghZ transcription direction of SG1.2 and SG1.5 are opposite to those of the remaining γ-PGA enzymes. The gene transcription of the *racE*, *pghZ* and γ-PGA synthetase gene clusters of N1142-3at, N1282-4at, N1108-5at and N1303-2Ay were opposite to the remaining γ-PGA enzymes.

The G2 genome contains other γ-PGA enzyme genes in addition to *pghZ*. With the exception of the SG2.3, SG2.5, and SG2.8 strains, the transcription directions of the *pghB*, *pghC*, *pghL* and *ggt* genes of the other G2 strains were opposite to those of the five genes in the γ-PGA synthetase gene cluster and *racE*. Notably, the base number distance from *pgdS* to *ywtC* on the γ-PGA synthetase gene cluster of strains 2RL2-3, HRBS-10TDI13, SRCM103886, GFR-12, 2KL1, and DKU_NT_03 is 1720 bp. The base number distance from *pgdS* to *ywtC* on the γ-PGA synthetase gene cluster of the SRCM101441 strain is 58,256 bp, so these strains are listed as the genotype 3. no *pghB* and *pghZ* genes in the genome of the G4 strain, and there were no *pghC*, *pghL*, *pghZ* and *ggt* genes in the genome of the G5 strain. The genome of the G6 genotype includes other γ-PGA enzyme genes except *pghL* and *pghZ*. The transcription directions of the *pghB*, *pghC*, and *pghL* genes of SG6.1 ∼ SG6.5 are the same, and the transcription directions of the γ-PGA synthase gene cluster and *racE* gene are opposite to that of the γ-PGA synthase gene cluster. The γ-PGA enzyme genes of SG6.1 and SG6.8 are in the same order on the genome, and the transcription direction of the *ggt* gene is opposite. Likewise, the γ-PGA genes of SG6.2 and SG6.9 are in the same order on the genome, and the transcription direction of the ggt gene is opposite, and the same is true for SG6.4 and SG6.10 strains. The type 7 strains lack the *ywtC* gene, and there are only three type 7 strains.

### Phylogenetic analysis

Through the research of evolutionary diversity of γ-PGA-related protein sequences, the possible phenotypic differences of producing γ-PGA among strains of the same genotype can be indicated.

Phylogenetic analysis based on the amino acid sequences of γ-PGA-related protein sequences from the isolated N3378-2at and N3378-3At strains as well as those from *Bacillus* spp. strains retrieved from GenBank indicated significant genetic variation among all γ-PGA hydrolases. Comparisons of the PgdS, PghB and PghC enzymes among *B. subtilis, B. velezensis* and *B. amyloliquefaciens* strains revealed amino acid identity ranges of 63–67%, 70–78% and 67.66–70.21%, respectively (Supplementary file 2). Remarkably, when compared to the GGT sequences of *B. subtilis* strains, the length and amino acid composition of GGT varies considerably in *B. velezensis* and *B. amyloliquefaciens* strains, as revealed by analysis.

**Fig. 4:**
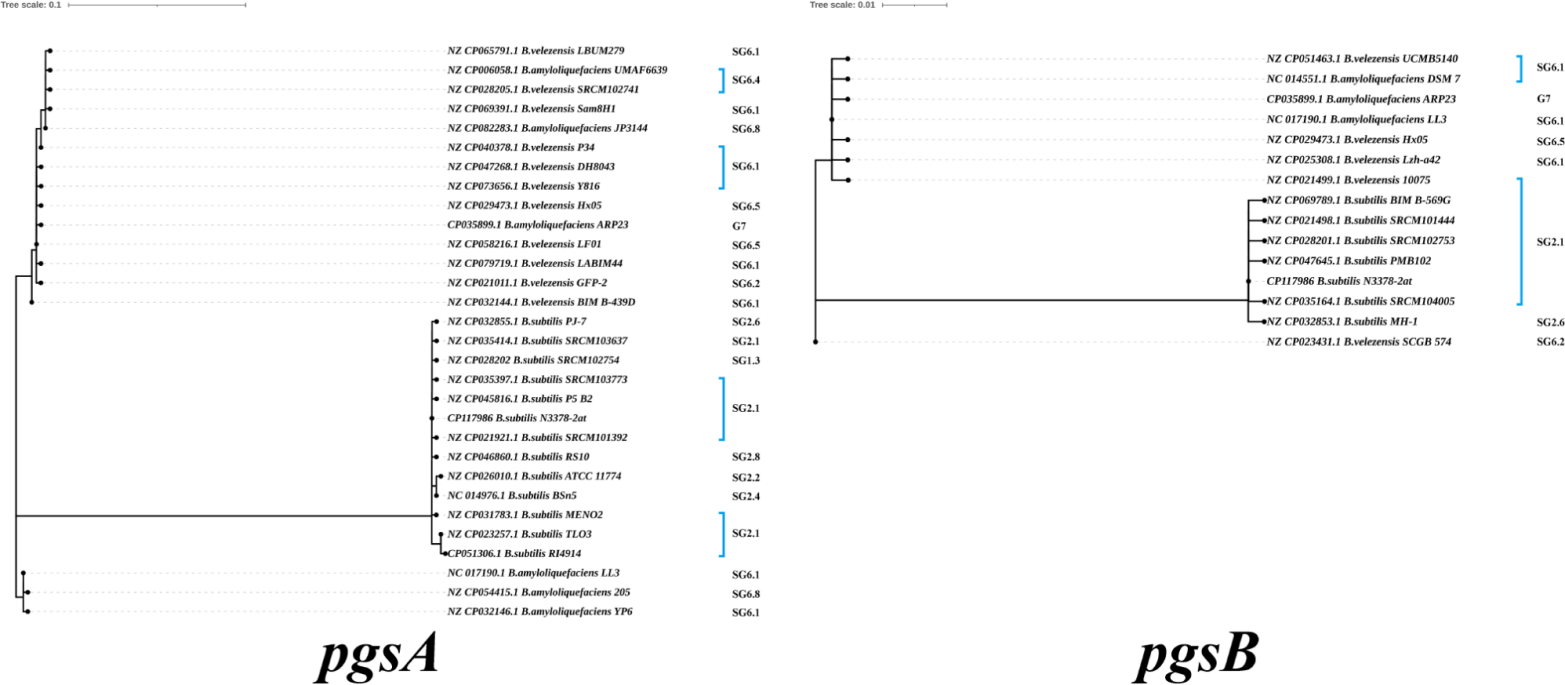
Phylogenetic trees of the amino acid sequences for the PgsA (**A**) and PgsB (**B**) of the *B. subtilis*, *B. velezensis* and *B. amyloliquefaciens* strains

The results indicated that 26 *B. subtilis* strains had mutations (D74N, E102D, K204N, G243E, V329I, and H362N) in PgsB relative to the N3378-2at strain, while 18 *B. velezensis* strains had mutations (K41Q, P239S, A265T, E280Q, V290I, and E381D), and 5 *B. amyloliquefaciens* strains had mutations (T170A and E381D) in PgsB relative to the *B. amyloliquefaciens* strain LL3. These sequence alignments suggested that these strains had an identity of 92.62 ∼ 93.13% at the amino acid level among *B. subtilis*, *B. velezensis*, and *B. amyloliquefaciens* strains. The PgsC protein had an identity of 92.62 ∼ 94.46% at the amino acid level. Therefore, compared to other γ-PGA-related enzymes, the PgsB and PgsC proteins displayed lower evolutionary divergence, but the PgsA evolutionary tree based on *B. subtilis* strains has 13 evolutionary branches, while the PgsA evolutionary tree of *B. velezensis* and *B. amyloliquefaciens* strains has 17 evolutionary branches (Fig. 4**).** The results showed that 47 *B. subtilis* strains harbored 13 mutations relative to the N3378-2at strain. The frequency of the E119K mutation was 3.73% (142/181), while the appearances of the other mutations were sporadic (below 1.7%). The *B. velezensis* and *B. amyloliquefaciens* strains harbored 18 mutations relative to the *B. amyloliquefaciens* LL3 strain. The T6S, N80K, E181Q, V191I, N233K and I349V mutations were the most frequent (93.07%, 93.51%), followed by the K90Q, A95S and K359D mutations, and the appearances of the other mutations were sporadic (below 1.7%). The sequence alignments indicated that PgsA had an identity of 78.01 ∼ 78.80% at the amino acid level among *B. subtilis*, *B. velezensis* and *B. amyloliquefaciens* strains. Compared to PgsB and PgsC, the PgsA protein displayed higher evolutionary divergence.

**Fig. 5:**
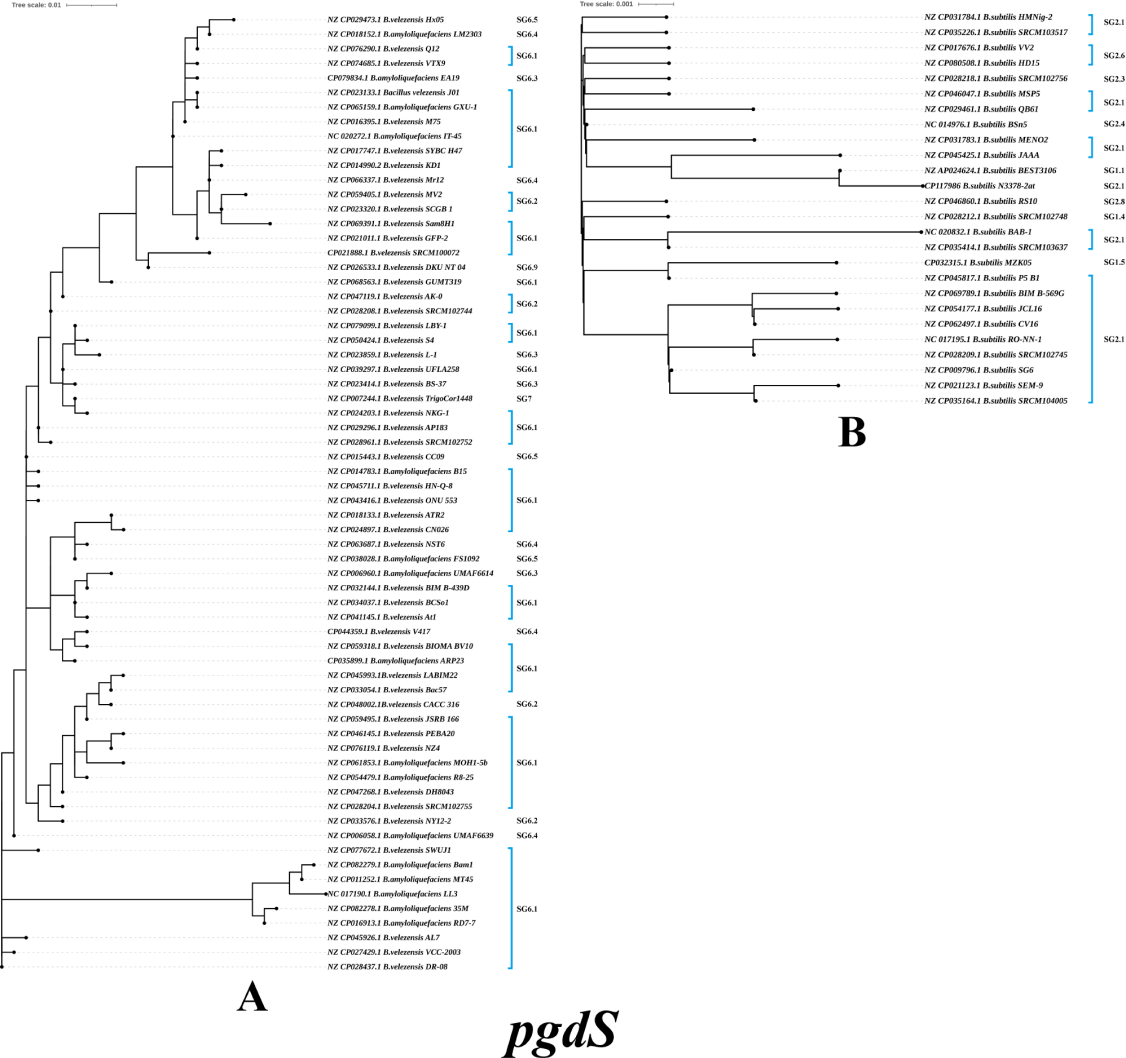
Phylogenetic trees of the amino acid sequences for the PgdS of *B. velezensis* and *B. amyloliquefaciens* strains (**A**), and *B. subtilis* strains (**B**)

The PgdS evolutionary tree based on *B. subtilis* strains has 27 evolutionary branches, while the PgdS evolutionary tree of *B. velezensis* and *B. amyloliquefaciens* strains has 70 evolutionary branches **(**Fig. 5A**)**. The sequence alignments indicated that 152 *B. subtilis* strains harbored 26 mutations relative to the N3378-2at strain. Interestingly, for *B. subtilis* strains, the PgdS protein was found to contain the highest frequency of mutant sites, including positions 112, 114, 224 and 354, when compared to the reference γ-PGA-producing strains examined in this study (Fig. 5B). The frequencies of the N112T, S114T, T224V and K354V mutations were 3.73% (142/181) and 0.13% (143/181), respectively, followed by the S36P (10/181), N45K (16/181) and K144Q (20/181) mutations, while the appearances of the other types of mutations were sporadic (below 5.5%). The *B. velezensis and B. amyloliquefaciens* strains harbored 70 mutations when compared to the *B. amyloliquefaciens* LL3 strain, and the frequencies of A36V, D55E, K58E, I67M, Q68H, E96D, I102L, L111T, T113N, Q117H, S140R, N145S, N166D, Y279F, Y303H, V328I, E342D, A349V, S352D, L385V and I410L mutations were higher than 80%, followed by the S227D, S227N, S227G, A263E, D293E, A313V and S407G (60–80%); the appearances of other types of mutations were sporadic. The sequence alignments indicated that they had an identity of 97.38–100% at the amino acid level among *B. subtilis* strains. *B. velezensis and B. amyloliquefaciens* strains had an identity of 89.2–100% at the amino acid level. Compared to the other γ-PGA-related enzymes, the PgdS protein exhibits considerable evolutionary variation among *Bacillus* sp. strains.

**Fig. 6:**
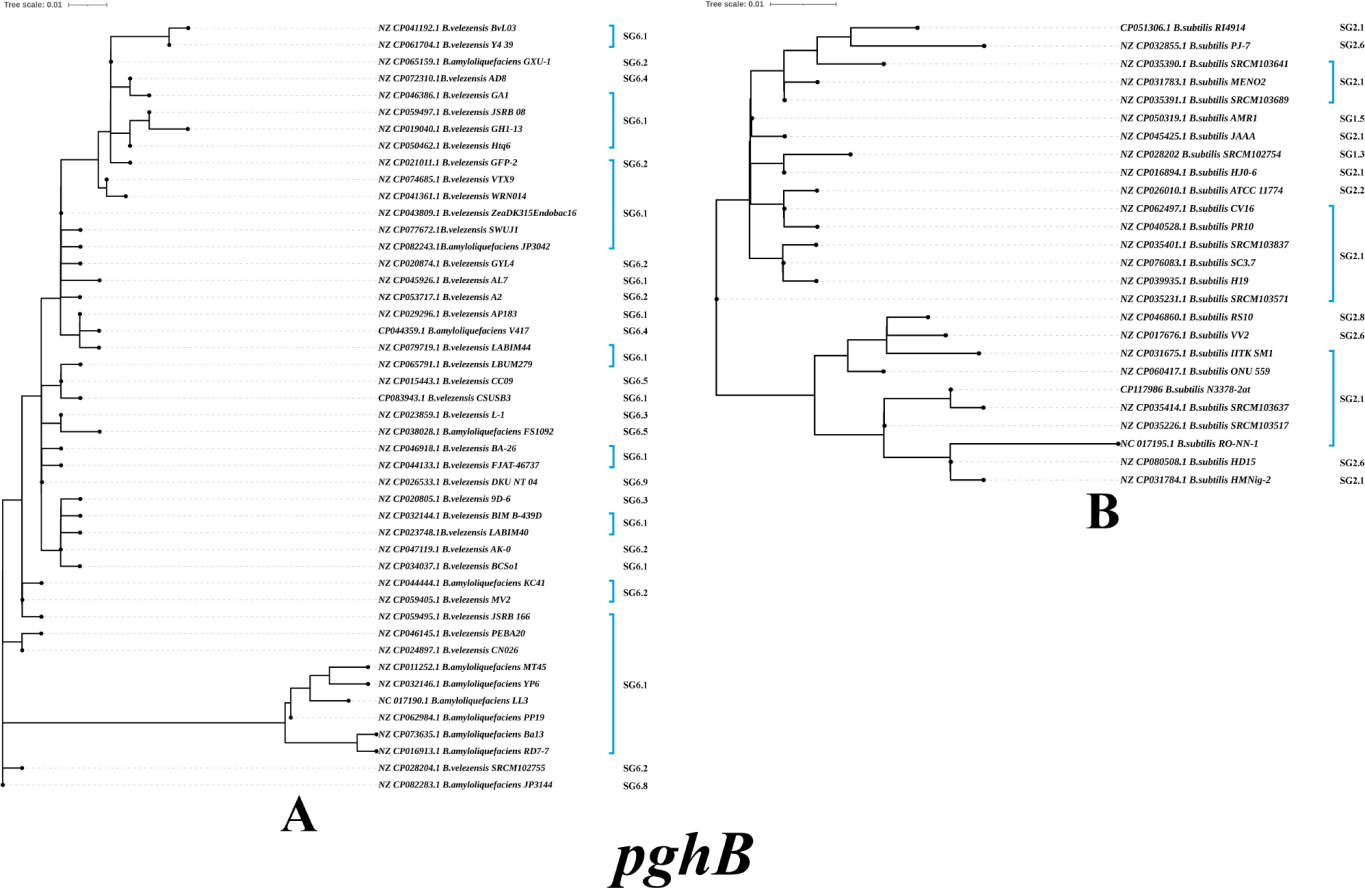
Phylogenetic trees of the amino acid sequences for the PghB enzyme of and the *B. velezensis* and *B. amyloliquefaciens* strains (**A**), *and B. subtilis* strains (**B**)

The PghB evolutionary tree based on *B. subtilis* strains has 26 evolutionary branches, while the PghB evolutionary tree of *B. velezensis* and *B. amyloliquefaciens* strains has 46 evolutionary branches (Fig. 6A). The *B. subtilis* strains harbored 25 mutations relative to the N3378-2at strain (Fig. 6B). The mutations with the greatest frequencies included K2E, V63L, A89D, N103K, S146N and C172R (65.03%, 77.05%, 71.04%, 76.50% and 66.67%, respectively), followed by K2T, M4R, L30F, Q65P and T200K (12.57%, 12.02%, 9.83%, 13.11% and 31.69%, respectively), and the appearances of the other types of mutations were lower than 26.96%. The *B. velezensis* and *B. amyloliquefaciens* strains harbored mutations relative to the LL3 strain, and the mutations with the greatest frequencies included K26E, Q65R, F70S, V84L, T104K, R119L, C130D, D135E, M136K, S168H, R178G, T180K and L196A (91.30%, 92.61%, 93.04%, 93.91% and 94.78%, respectively), followed by S7N, E29K, N67D, A93G and E200D (79.57%, 76.09%, 77.83% and 41.30%, respectively); the frequencies of other types of mutations were lower than 26.96%. *B. velezensis* and *B. amyloliquefaciens* strains harbored 48 mutations when compared to the *B. amyloliquefaciens* LL3 strain. The mutations with the greatest frequencies included K26E, Q65R, F70S, V84L, T104K, R119L, C130D, D135E, M136K, S168H, R178G, T180K and L196A (91.30%, 92.61%, 93.04%, 93.91% and 94.78%, respectively), followed by the S7N, E29K, N67D, A93G and E200D (79.57%, 76.09%, 77.83% and 41.30%, respectively), and the frequencies of other types of mutations were lower than 2.2%. An identity of 92.50–100% was obtained at the amino acid level among *B. subtilis* strains based on sequence alignments. *B. velezensis* and *B. amyloliquefaciens* strains had an identity of 87.06–100% at the amino acid level. The PghB protein has displayed considerable evolutionary variation among *Bacillus sp.* strains compared to the other γ-PGA-related enzymes.

The PghC enzyme sequences among *B. subtilis*, *B. amyloliquefaciens* and *B. velezensis* strains from isolates and GenBank were listed and analyzed. As shown in Supplementary Fig. 7A, the PghC evolutionary tree based on *B. subtilis* strains has 8 evolutionary branches, while the PghC evolutionary tree of *B. velezensis* and *B. amyloliquefaciens* strains has 23 evolutionary branches. These sequence alignments indicated that they had an identity of 99.15 ∼ 100% at the amino acid level among *B. subtilis* and an identity of 83.82 ∼ 100% at the amino acid level among *B. amyloliquefaciens* and *B. velezensis* strains. The PghC of *B. subtilis* strains harbored 8 mutations when compared to the N3378-2at strain, the most frequent mutation was L235S (88.52%), and the appearances of the other mutations were sporadic (below 4.37%). The *B. velezensis* and *B. amyloliquefaciens* strains harbored 36 mutations when compared to the *B. amyloliquefaciens* LL3 strain. The most frequent mutations included V21A, Q58R, E59K, S62P, F88Y, L115S, K118E, R124H, S134G, K154R, R169K, S173A, I177T, T184A and Y222F (93.04, 93.91, 91.74 and 94.35%, respectively), followed by T98A and K157E (44.35 and 65.22%, respectively), and the appearances of the other mutations were sporadic (below 17.39%).

**Fig. 7:**
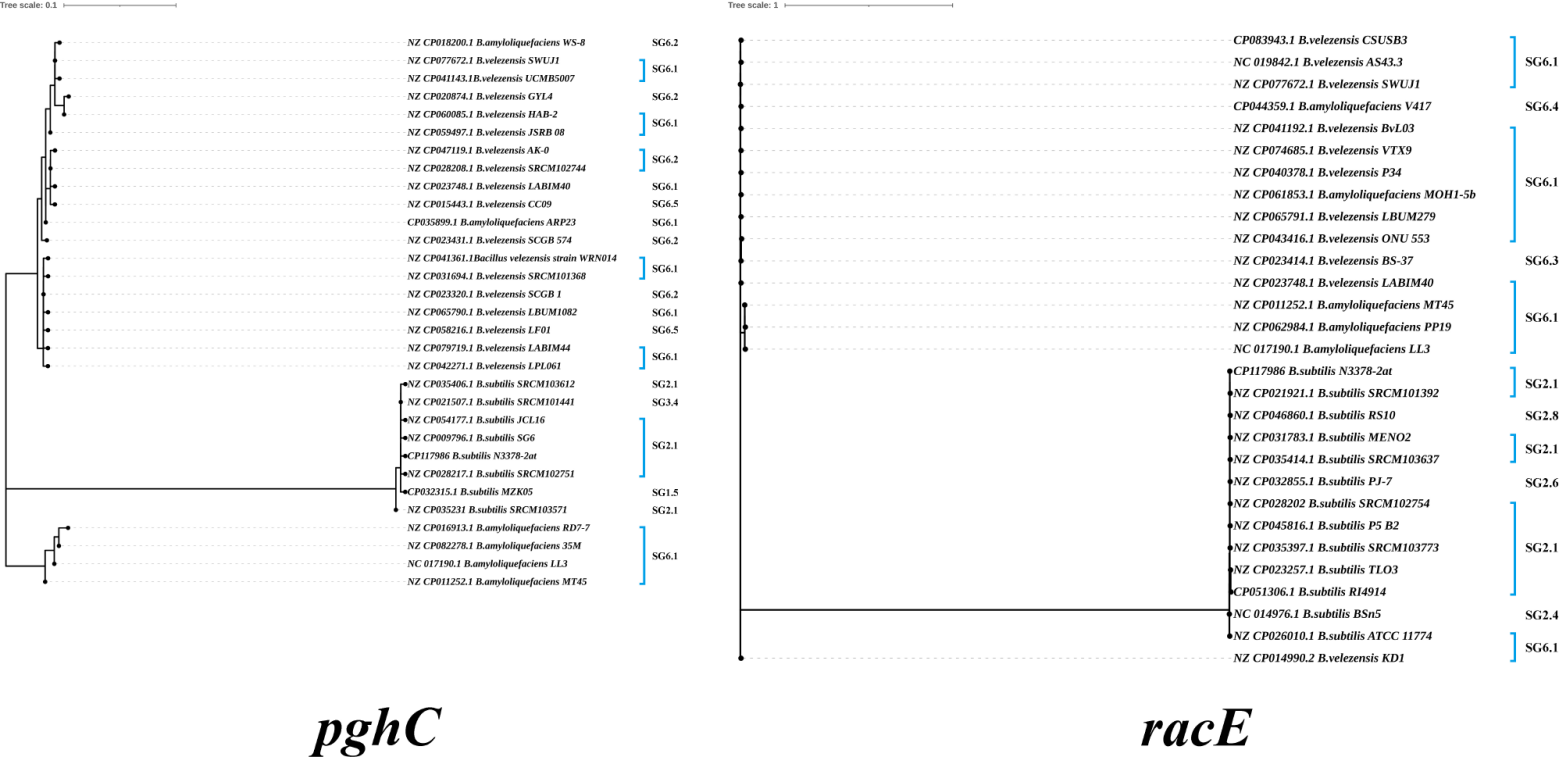
Phylogenetic trees of the amino acid sequences for the PghC (**A**) and RacE (**B**) of the *B. velezensis* and *B. amyloliquefaciens* strains

The RacE enzyme sequences among *B. subtilis*, *B. amyloliquefaciens* and *B. velezensis* strains from isolates and GenBank were listed and analyzed. As shown in Fig. 7B, the RacE evolutionary tree based on *B. subtilis* strains has 13 evolutionary branches, while the RacE evolutionary tree of *B. velezensis* and *B. amyloliquefaciens* strains has 15 evolutionary branches. These sequence alignments indicated that they had an identity of 83.82 ∼ 100% at the amino acid level among *B. velezensis* and *B. amyloliquefaciens* strains. The RacE of *B. subtilis* strains harbored 11 mutations when compared to the N3378-2at strain, and the most frequent mutations included P230A, I231N and K271R (36.61% and 37.15%, respectively), while the appearances of the other mutations were sporadic (below 3%). The *B. velezensis* and *B. amyloliquefaciens* strains harbored 18 mutations when compared to the *B. amyloliquefaciens* LL3 strain. The most frequent mutation included I119V, D137E, A177S, D197N, K201E and I270V and M143T (93.51% and 97.84%), while the appearances of the other mutations were sporadic (below 3.03%). The sequence alignments indicated that RacE had an identity of 83.82 ∼ 85.29% at the amino acid level among *B. subtilis*, *B. velezensis* and *B. amyloliquefaciens* strains. Thus, RacE displayed lower evolutionary divergence than that of the other γ-PGA-related enzymes.

## Discussion

In the field of life sciences, Bacillus natto currently serves as the primary vector for industrial production of γ-PGA, a polymer of high commercial interest due to its biodegradable nature and multiple industrial applications. Although supplementing more L-glutamic acid can enhance γ-PGA production, this approach amplifies the production costs significantly. Consequently, the use of glutamate-independent strains is becoming the preferred choice for industrial production, owing to their cost-effectiveness and streamlined production processes [44].

The biochemical structure of γ-PGA, characterized by the linkage through γ-amide bonds, confers resistance against most common proteases. However, this polymer can be degraded by specific hydrolases, primarily found in *Bacillus* spp [16]. . Notably, there are two key γ-PGA-degrading enzymes in this genus. The first is PgdS that exclusively cleaves the γ-glutamyl bond linking D-glutamic and L-glutamic acid in γ-PGA [21–23]. The second is γ-glutamyl transpeptidase (GGT) which, despite its lack of connection to the *pgs* operon, plays a pivotal role in γ-PGA degradation. Under adverse environmental conditions, such as nitrogen limitation during starvation, GGT provides constituent glutamates as a nutrient source by breaking down γ-PGA [5, 6]. Interestingly, these enzymes can also secrete into the surrounding medium and degrade the high molecular weight γ-PGA into smaller fragments ranging from 1 to 20 kDa [45].

Additionally, γ-PGA hydrolases sourced from phages pose significant challenges in modern natto production facilities, compromising the desirable viscosity characteristic of natto products [7]. Taking this into consideration, Kimura and colleagues successfully isolated a phage, termed NIT1, from a natto product and infected *B. subtilis* natto to further study the phage-derived γ-PGA hydrolase [46]. PghP, a 23-kDa metallopeptidase, requires Zn^2+^ for its activity and degrades high molecular weight γ-PGA [47, 48]. The discovery of four previously unannotated *B. subtilis* genes (encoding YjqB, YmaC, YndL and YoqZ) exhibiting high resemblance to PghP (with 27–37% identity and 41–54% homology) has also been reported. Given their location in prophagic regions of the *B. subtilis* 168 genome, it can be surmised that these genes likely stem from integrated prophages and are denominated as *pghB*, *pghC*, *pghL* and *pghZ* [7].

Further, we have discovered two new γ-PGA-producing *B. subtilis* strains from natto samples, namely N3378-2at and N3378-3At. The total yield of γ-PGA production was lower in the glutamic acid-independent strain (1.6328 g/L for N3378-3At) than its counterpart (2.0568 g/L for N3378-2at). In order to ascertain the molecular evolutionary mechanism of γ-PGA-associated enzymes, an in-depth analysis was conducted, focusing primarily on the identification of enzymes previously reported influencing γ-PGA production in the genomes of these two strains. This investigation revealed various critical markers, leading to the identification of the γ-PGA synthetase gene cluster (comprising PgsB, PgsC, PgsA, YwtC, and PgdS), glutamate racemase RacE, phage-derived γ-PGA hydrolase (with representatives PghB, PghC, PghL, and PghZ), and exo-γ-glutamyl peptidase (GGT) in the genomes of N3378-2at and N3378-3At. Subsequent examination of 183 *B. subtilis*, 55 *B. amyloliquefaciens*, and 175 *B. velezensis* strains revealed the presence of these γ-PGA-related enzymes across all strains except for two *B. subtilis* and three *B. amyloliquefaciens* strains. Notably, the prophagic gene products *pghL* and *pghZ* were exclusively found in *B. subtilis* genomes (181 and 57 hits, respectively).

Each gene within the *pgsBCA* cluster a play a pivotal role in γ-PGA production and metabolism [19]. Specifically, *pgsB* is essential for peptide synthase activities, which are responsible for γ-PGA synthesis, whereas *pgsC* likely enhances precursor supply by transferring γ-glutamyl units to *pgsB* [44]. Concurrently, *pgsA* is believed to regulate γ-PGA production or export [2]. The deglutamylase activity of pgdS, by inducing degradation, controls intracellular γ-PGA levels, while transport functions are largely governed by *pghC*, *pghB*, *pghL*, and *pghZ*, all of which encourage efficient transfer of γ-PGA across the bacterial cell wall and membrane [24]. Additionally, *ywtC*, *racE*, and *ggt* perform integral roles in γ-PGA metabolism by engaging in processes such as AMP-binding, L-glutamate provisioning, and hydrolysis, respectively [17, 20, 21]. Genotypes SG2.2 and SG2.1 were identified in *B. subtilis* N3378-2at and N3378-3At strains, respectively, while non-γ-PGA-secreting *B. subtilis* strains appertained to the G1 genotype (Fig. 3). The genomic positioning of these genes seemingly exerts a significant influence on their expression profiles, which could subsequently influence the production efficiency of γ-PGA to a certain degree. This is because proximity to the replication origin prescribes the timing of gene duplication during replication, potentially leading to higher gene copy numbers in rapidly proliferating cells [49–51]. As a result, the genomic placement of these genes may heavily influence the speed and efficacy of γ-PGA production in *B. subtilis*. However, the precise mechanics behind these positional effects are not fully understood, necessitating further research.

Upon performing a comparative analysis on the amino acid sequences from γ-PGA-related enzymes, significant disparities, chiefly in PgdS, PghB, PghC, and GGT, were revealed. This suggests a potential link between sequence heterogeneity and enzymatic functionality, a proposition warranting thorough experimental investigation. Advanced research should focus on determining whether specific amino acid variations in γ-PGA-associated hydrolases manipulate enzymatic activity, thereby impacting γ-PGA yields. Such findings could potentially guide bioengineering strategies for enhancing industrial γ-PGA production. Furthermore, examining the positional effects of genes within the γ-PGA synthesis pathway and evaluating the contributions of prophage-derived genes to the metabolism and accumulation of γ-PGA can engender a more profound comprehension of the genetic and regulatory subtleties that shape γ-PGA biosynthesis in *Bacillus* spp. Exploiting the genetic pliability of *B. subtilis* offers promising prospects for engineering the system to optimize γ-PGA production.

## Conclusion

In conclusion, we have isolated two γ-PGA-producing strains *B. subtilis* N3378-2at and N3378-3At from natto, and their yield of γ-PGA have reached 2.0568 g/L and 1.6328 g/L, respectively. From their genomes, we have identified γ-PGA-related protein sequences. Based on these protein sequences, we carried out genotyping analysis and classified 183 *B. subtilis* into genotypes 1–5, 175 *B. velezensis* and 55 *B. amyloliquefaciens* strains were classified into genotype 6 and 7. Furthermore, PgsB, PgsC, PgsA and RacE were relatively conserved, while PgdS, PghB, PghC and GGT showed genetic diversity among *B. subtilis*, *B. amyloliquefaciens* and *B. velezensis* strains.

### Electronic supplementary material

Below is the link to the electronic supplementary material.

Supplementary Material 1

Supplementary Material 2

Supplementary Material 3

## Data Availability

The sequence data and details of the sequenced samples, including the date and location of the collection and source, were submitted to the GenBank: N3378-2at (CP117986, https://www.ncbi.nlm.nih.gov/search/all/?term=CP117986), N3378-3At (CP125812, https://www.ncbi.nlm.nih.gov/search/all/?term=CP125812). All data generated or used during the study appeared in the submitted/supplementary article.
