# Supplementary material for: Genomic characterization and related functional genes of γ- poly glutamic acid producing Bacillus subtilis
Source: BMC Microbiol. 2024 Apr 15;24:125. doi: 10.1186/s12866-024-03262-z (PMC11017564; doi:10.1186/s12866-024-03262-z)

Supplementary materials

Identification, gene typing and sequencing analysis of functional genes of poly-γ-glutamic acid secreting *Bacillus subtilis* N3378-2at and N3378-3At strains

Jiayue Zhu^1†^, Xue Wang^2†^, Jianan Zhao^2^, Fang Ji^2^, Jun Zeng^2^, Yanwen Wei^2^, LiLi Xu^3*^, Chengmin Wang^2*^ ,Xingyuan Ma^1*^

^1^State Key Laboratory of Bioreactor Engineering, East China University of Science and Technology, Shanghai, China

^2^Guangdong key Laboratory of Wild Animal Conservation and Utilization, Institute of Zoology, Guangdong Academy of Science, Guangzhou, China.

^3^Union Biology (Shanghai) Co., Ltd, Shanghai, China.

**Supplementary figures legends**

**FIGURE** **1 |** Phylogenetic trees of the amino acid sequences for the PgsA and PgsB of the *B. subtilis*, *B. velezensis* and *B. amyloliquefaciens* strains.


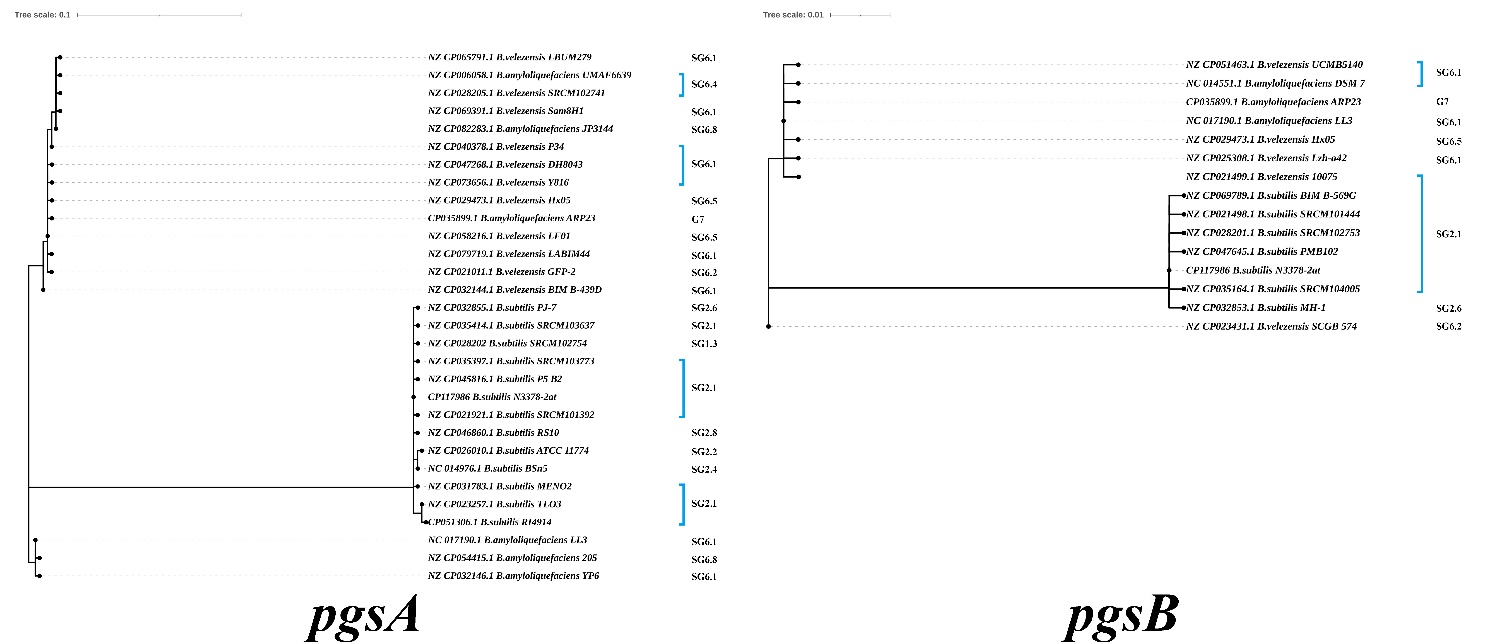


**FIGURE** **2 |** Phylogenetic trees of the amino acid sequences for the PgdS of the *B. subtilis* strains and the B. velezensis and B. amyloliquefaciens strains.

.


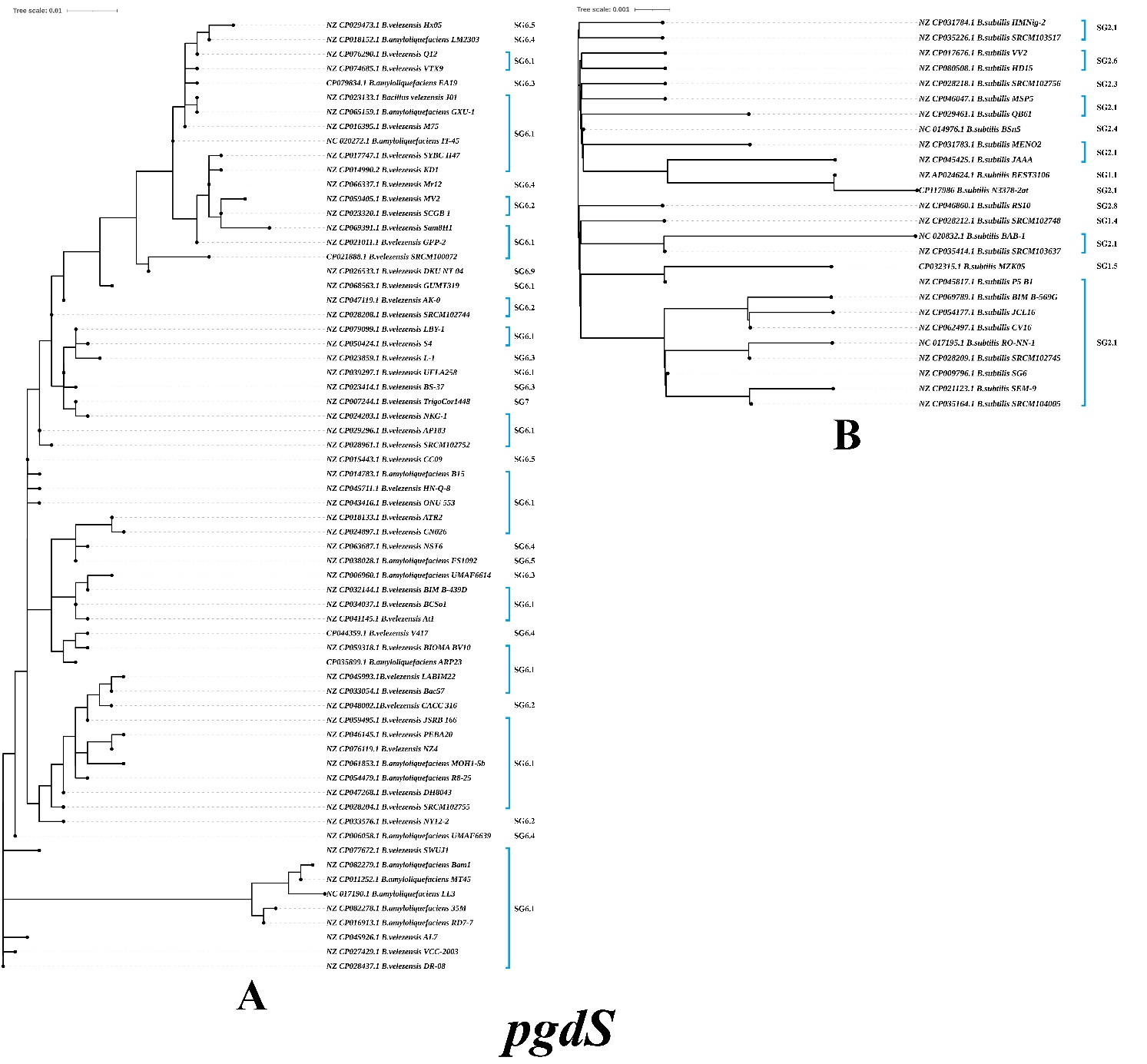


**FIGURE 3 |** Phylogenetic trees of the amino acid sequences for the PghB enzyme of the *B. subtilis* strains and the *B. velezensis* and *B. amyloliquefaciens* strains


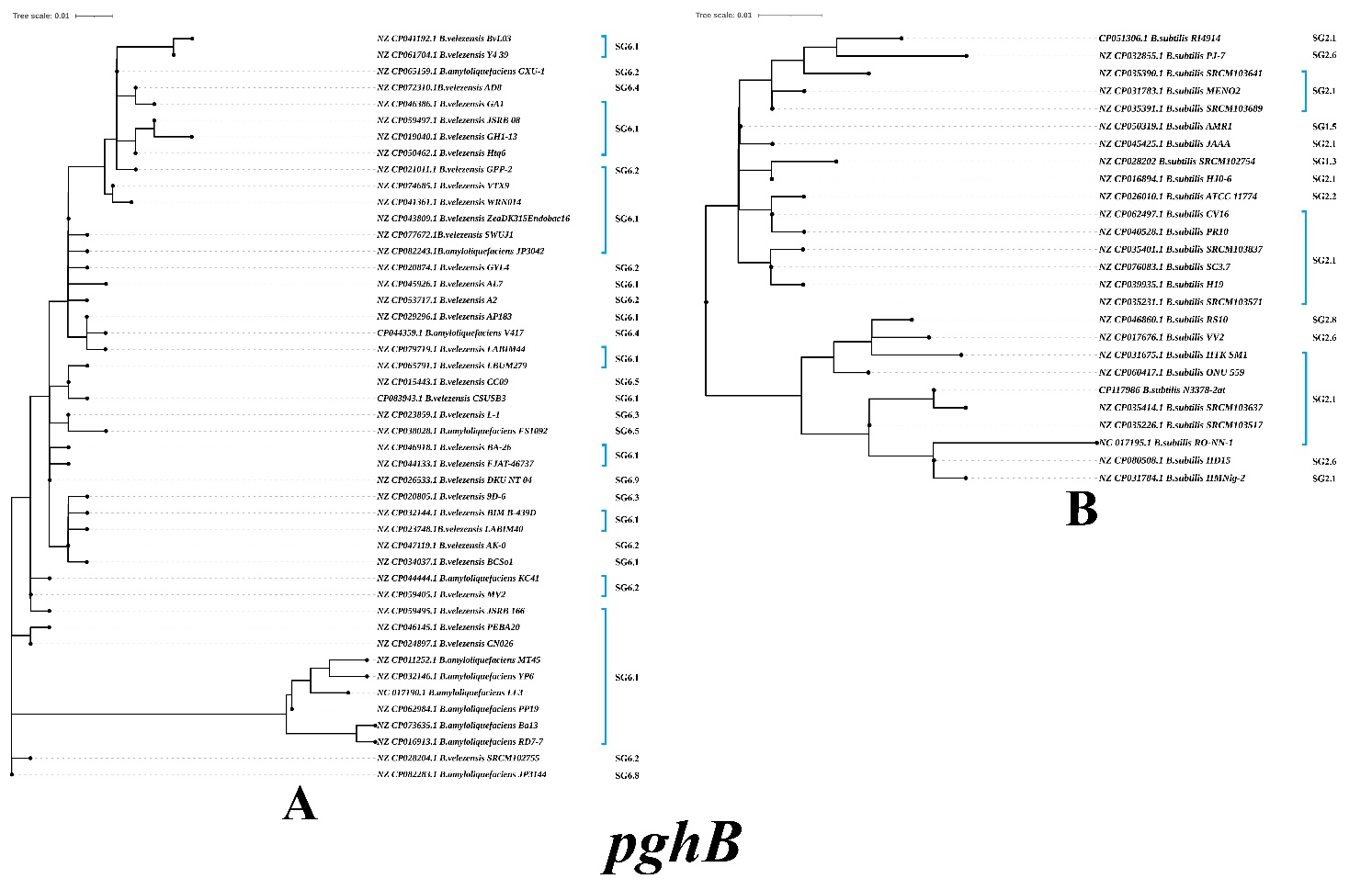


**FIGURE 4 | Phylogenetic trees of the amino acid sequences for the PghC and** RacE **of the *B. velezensis* and *B. amyloliquefaciens* strains.**


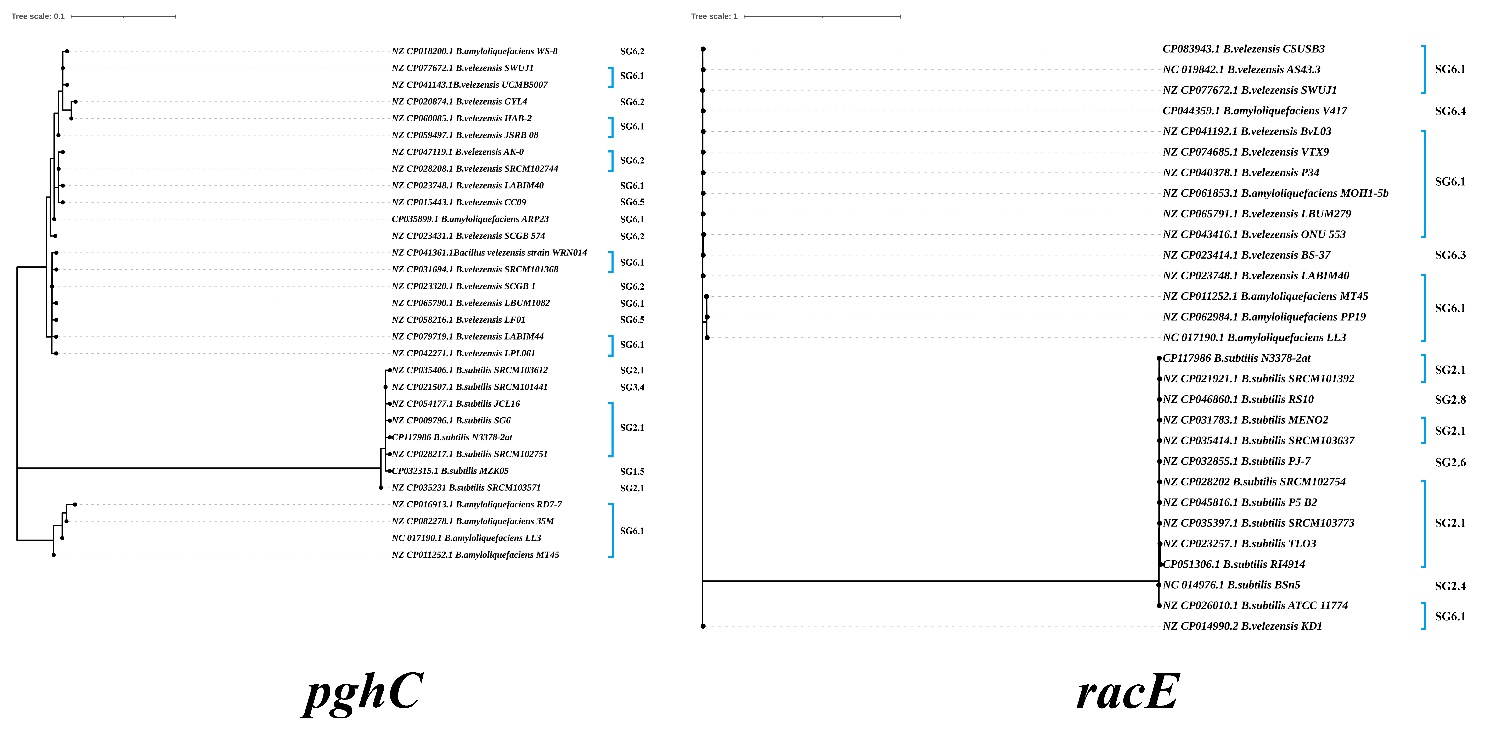

Supplement: Supplementary file 3 — Supplementary Material 3 [file 12866_2024_3262_MOESM3_ESM.docx]
